# Mechanisms of GPM6A in Malignant Tumors

**DOI:** 10.1002/cnr2.70137

**Published:** 2025-02-17

**Authors:** Bei Huang, Xihong Li

**Affiliations:** ^1^ Operation Management and Evaluation Department, West China Second University Hospital Sichuan University Chengdu Sichuan People's Republic of China; ^2^ Key Laboratory of Birth Defects and Related Diseases of Women and Children (Sichuan University) Ministry of Education Chengdu Sichuan People's Republic of China; ^3^ Emergency Department, West China Second University Hospital Sichuan University Chengdu Sichuan People's Republic of China

**Keywords:** colorectal cancer, glioblastoma, GPM6A, liver cancer, lung cancer

## Abstract

**Background:**

Glycoprotein M6A (GPM6A) encodes a transmembrane protein, expressing in large quantities on the cell surface of central nervous system (CNS) neurons. GPM6A acts importantly in neurodevelopment by modulating neuronal differentiation, migration, axon growth, synaptogenesis, and spine formation, but its role in malignancy remains controversial and requires further research. This article reviewed the mechanisms of GPM6A in colorectal cancer, liver cancer, lung cancer, glioblastoma, and other malignant tumors, and made a “one‐stop” summary of the relevant mechanisms.

**Recent Findings:**

Researches have indicated that GPM6A is related to malignant tumors. It affects epithelial‐mesenchymal transition and induces the formation of filopodia, participating in the adhesion, migration, and metastasis of cancer cells. Its role in malignant tumors remains controversial, however. On the one hand, GPM6A may have carcinogenic properties and is related to poor prognosis of malignant tumors. It is highly expressed in lymphoblastic leukemia and is a potential oncogene. It also shows carcinogenic properties in colorectal cancer, glioblastoma, gonadotroph adenomas and so on. On the other hand, the expression of GPM6A decreases in lung adenocarcinoma, liver cancer, thyroid cancer, and so forth as the tumor progresses, and it can inhibit the progression of malignant tumors by inhibiting some signaling pathways, suggesting that it may be a tumor suppressor gene.

**Conclusion:**

Carcinogenic or tumor suppressive? Although the biological function of GPM6A in the development of malignant tumors is still unclear, according to the current research progress, it is still expected to become an effective molecular marker for predicting tumor occurrence, metastasis and prognosis, as well as a new target for diagnosis and treatment.

## 1 Introduction

Glycoprotein M6A (GPM6A) is highly expressed in the neurons of central nervous system (CNS) [1, 2]. The protein encoded by *GPM6A* gene belongs to the proteolipid protein (PLP) family, which is considered as the main constituent of the myelin sheath of brain [3] and is suspected to be housekeeping protein involved in intracellular transport [4]. Researches have shown that GPM6A acts importantly in neurodevelopment by modulating neuronal differentiation [5, 6], migration [6], axon growth [7, 8], spine formation [7], and synaptogenesis [9]. In the hippocampus of patients who commit suicide due to depression, the transcription of GPM6A and GPM6B is downregulated [10]. Chronic physical and social stress reduces levels of GPM6A mRNA in hippocampus, while the antidepressant drug tianeptine prevents this downregulation [11]. The *GPM6A* gene is disordered in people with schizophrenia, and may play a role in it via making effect on brain development [12]. Altering the dose of GPM6A/M6 impairs cognitive ability in humans and fruit flies [13]. In mutant mice with GPM6A deletion, mild stress induces a claustrophobic phenotype [14]. Abundant genomic and proteomic researches have found that the molecular structure or expression level of GPM6A is altered in a variety of neuropsychiatric disorders.

Recently, researches have indicated that it is also related to malignancy, and its role in malignancy is controversial. GPM6A and glycoprotein M6B (GPM6B) may have carcinogenic properties [3]. In lymphoid leukemia, GPM6A is abundantly expressed. It promotes the proliferation and transformation of fibroblasts NIH/3T3, making it a potential oncogene for lymphoid leukemia [3]. Protein tyrosine phosphatase receptor type Z1 (PTPRZ1) and GPM6A are essential for the formation of glioblastoma (GB) stem cells (GBSC) spheres. Blocking either PTPRZ1 or GPM6A may be a method for the treatment of GB [15]. Decreased GPM6A expression was found in highly differentiated colorectal cancer (CRC) tissues, while higher expression levels were observed in minimally differentiated or undifferentiated colon cancer tissues [16]. However, GPM6A inhibits lung adenocarcinoma progression via inhibiting phosphoinositide 3‐kinase (PI3K)/AKT serine (AKT) pathways [17]. Overexpression of miR‐22 inhibits the migration of small‐cell lung cancer (SCLC) cells, and GPM6A was found to be increased in cells with overexpression of miR‐22 [18]. The levels of GPM6A found in hepatocellular carcinoma (HCC) tissue are much lower than those in pericancerous liver tissue, and the oncogenic microRNA‐96 function is significantly suppressed in liver cancer cells with overexpression of GPM6A [19]. GPM6A expression in normal samples is significantly higher than that in malignant tumor samples, indicating that GPM6A is inhibited in malignant tumors. Through PubMed, Medline, and Web of Science, this narrative review searched all the studies on GPM6A and tumors/cancers up to September 2024, and summarized the role of GPM6A in CRC, lung cancer, liver cancer, GB, and other malignant tumors, and made a summary of the relevant mechanisms for further researches.

## 2 GPM6A Gene and Protein

*GPM6A* is a member of the proteolipid protein gene family. It is highly expressed in CNS neurons [1, 2]. Located on chromosome 4q34.2, human *GPM6A* gene consists of 7 exons and its total length is 369 731 kb [20].

GPM6A was discovered in 1992 [21, 22], and is expressed in large quantities on the surface of CNS neurons [1], especially in the hippocampus, prefrontal cortex, and cerebellum [23]. GPM6A is a tetraspanin [24] composed of 278 amino acids, with a molecular mass of about 32 kDa [20]. GPM6A molecules have the classical structure of PLP family members: four transmembrane domains (TMDs), one intracellular loop (IC), two extracellular loops EC1 and EC2, and the cytoplasm‐oriented N‐ and C‐terminal regions [25]. TMDs are highly conservative [26]. Among the tetraspanins with similar structure, the second large extracellular ring EC2 determines functional specificity, and the cytoplasmic tail plays the role of connecting with cytoskeleton or signal protein [27]. EC2 has four cysteine residues, playing important roles in its folding and function. Among them, a disulfide bond connects C174 to C192, forming an important domain for protein–protein interactions [9]. The EC2 region contains cysteine residues, which is involved in disulfide bond formation. Disulfide bonds are crucial for the domain structure and critical for the role of proteins in filopodia growth [9]. The EC2 domain of GPM6A also contains two potential *N*‐glycosylation sites [9], and the *N*‐terminal intracellular domain is necessary for axon growth arrest [28]. Monoclonal antibodies recognizing cell surface epitopes only recognize EC2 [29]. EC1 domain is required for protein expression on the correct surface, and is responsible for the binding strength of EC2 interactants [30]. Therefore, the function of GPM6A molecules can be explored by studying the protein molecules interacting with the extracellular ring. Using bioinformatics to analyze the single nucleotide polymorphisms (SNPs) of human GPM6A, 13 high‐risk mutants that have an important impact on the structure and function of GPM6A were found, which will affect the correct folding of proteins, leading to loss of function and disease [31].

## 3 Physiological Function of GPM6A

There is little research on the relationship between the molecular structure of GPM6A and its physiological functions at present. Current research suggests that GPM6A plays a role in the signal transduction of lipid rafts during neuronal differentiation [32, 33, 34], promotes neuronal polarization and axon formation [23, 28, 34], and induces the formation of filopodia [33] and dendritic spines [35] (Table 1). However, the biological function of GPM6A in the occurrence and development of malignant tumors is not well understood.

**TABLE 1:** Physiological functions of GPM6A.

First author, year	Species	Functions
Nozumi et al., 2017	Rats, mice	Participating in signal transduction of lipid rafts during neuronal differentiation [32, 33, 34].
Scorticati et al., 2011	Rats	
Honda et al., 2017	Mice	
Cooper et al., 2008	Rats, mice	Promoting the polarization of neurons and the formation of axons [23, 28, 34].
Sato et al., 2011	Armenian hamsters, mice	
Honda et al., 2017	Mice	
Scorticati et al., 2011	Rats	Inducing the formation of filopodia [33] and dendritic spines [35].
Gu et al., 2018	Mice	
Zhang et al., 2022	Human, mice	Downregulation of GPM6A enhanced EMT of lung adenocarcinoma cells and vice versa [17].

Abbreviation: EMT, epithelial‐mesenchymal transition.

Metastasis is a typical feature of malignant tumors and also a major cause of high mortality in most malignancies [36]. Metastasis is highly complex and involves different cellular mechanisms, including primary tumor division, evasion of immune surveillance, invasion, and regulation of tissue microenvironment [37]. Being necessary for most malignant tumor metastasis and spread [38, 39], epithelial‐mesenchymal transition (EMT) is a step‐wise process leading to a switch from epithelial to mesenchymal phenotype [40]. It plays a crucial role in tumor invasion, and it is a way for cancer cells to acquire more aggressive characteristics [41]. As a biomarker of EMT [42], Zinc finger E‐box binding homeobox 1 (ZEB1) gene promotes EMT through signals such as ZEB1‐ERK/MAPK [43], transforming growth factor beta (TGF‐β)/Smad [44], lnc‐Nr2f1 [45], and various signal axes, including pro‐transition associated RNA (PTAR)‐miR‐101‐ZEB1 [46], LncRNA XIST/miR‐101‐3p/ZEB1 [47], reactive oxygen species (ROS)/miR‐200c/ZEB1 [48] and ZEB1/YAP1‐ITGA3 [49]. GPM6A is positively correlated with ZEB1 in GB [15]. Upregulation of the fat mass and obesity‐associated protein (FTO) further enhances the expression and stability of ZEB1 transcripts through reducing N6‐methyladenosine (m6A) RNA methylation, resulting in chemotherapy resistance and EMT in tumor cells [50]. When analyzing the expression of GPM6A mRNA in cancer using The Cancer Genome Atlas (TCGA) dataset, it was found that downregulation of GPM6A enhanced the migration, proliferation, and EMT of lung adenocarcinoma cells, whereas upregulation of GPM6A inhibited their development [17]. Unlike TGF‐β, E‐cadherin, and N‐cadherin, the role of GPM6A in EMT is not very clear. EMT directed by TGF‐β drive cancer progression [51]. During EMT, the decreased expression of E‐cadherin is paralleled with an increased expression of *N*‐cadherin [40]. In GB, GPM6A is positively correlated with ZEB1, which promotes EMT, while in lung adenocarcinoma, downregulation of GPM6A can enhance EMT. Therefore, the relationship between GPM6A and EMT requires further investigation.

Filopodia are necessary for cells to infiltrate into tissues and eventually complete effective metastasis [52]. As a sensory organ of cells, filopodia receive signals such as chemical attractants and nutrients [53], and play an important role in neurite growth, cell migration, and wound healing, among which cell migration is the most characteristic [54]. Rich filopodia are related to enhanced metastasis and invasion of malignant tumor cells [55, 56, 57]. It contributes to intercellular communication [58], local invasion [59], directed migration [59, 60, 61, 62] and cell adhesion [63, 64, 65], and is a prominent feature of aggressive or migratory malignant tumor cell. In breast malignant tumor cells, actin polymerization and filopodia are mediated by C‐C chemokine receptor 7 (CCR7) or C‐X‐C chemokine receptor type 4 (CXCR4) signaling, which subsequently induces chemotactic and invasive responses [66]. The reduction of filopodia reduces tumor invasion and metastasis [67]. Besides, filopodia and lamellipodia are interchangeable [53]. As the main organelle of cell movement [53], lamellipodia are mainly responsible for the long‐distance migration of cells [68], and is crucial for the metastasis of malignant tumor cells [69]. Filopodia are the main driving force for cells to move forward, while lamellipodia help cells establish connections with their surroundings and provide stable support. Both work together to ensure that cells can effectively migration and metastasis within the microenvironment [70]. GPM6A in zebrafish and rats has been shown to induce the formation of filopodia [9, 71, 72]. Exogenous overexpression of GPM6A and GPM6B in NIH/3T3 cells alters microtubule networks and actin, and induces long filopodia‐like protrusion formation [3]. GPM6A accumulates in the lipid raft domain in normal neuron cells, acting as a transducer of extracellular signals like laminin [73]. Palmitoacylated GPM6A localized in lipid rafts can promote the formation of filopodia through its downstream signaling molecules, but GPM6A detached from lipid rafts cannot induce filopodia formation [33]. In cultured rat hippocampal neurons, phosphorylation of GPM6A localized to lipid rafts promotes filopodia formation [72]. C174 and C192 of the second extracellular ring of GPM6A are important in the growth of filopodia and synaptogenesis [9]. Mutations of glycine in TMD2 and TMD4 affect the formation of dimers and prevent them from inducing the formation of filopodia [74, 75]. The *C*‐terminal amino acid residues E258, K255, and K250 are also of significant importance for filopodia formation induced by GPM6A [25]. The kinase activity of p21‐activated kinase 1 (PAK1) is required for GPM6A‐induced filopodium formation, and PAK1 is a main downstream effector of ras‐related C3 botulinum toxin substrate 1 (RAC1) which acts downstream of the actin regulatory protein coronin‐1a (CORO1A) [76]. In rat hippocampal neuron models cultured in vitro, the endogenous GPM6A specifically associates with CORO1A, and CORO1A activate its downstream PAK1 and RAC1 signaling pathways, promoting the formation of filopodia [76]. Inhibition of Src kinase and mitogen‐activated protein kinase (MAPK) prevents filopodia formation in M6a‐overexpressed neurons [33] (Table 2).

**TABLE 2:** GPM6A is involved in filopodia formation in various conditions.

First author, year	Conditions	Functions in filopodia formation
Scorticati et al., 2011	Palmitoacylated GPM6A localized in lipid rafts	Promote filopodia formation through its downstream signaling molecules [33].
Brocco et al., 2010	Phosphorylation of GPM6A localized to lipid rafts	Promote filopodia formation [72].
Formoso et al., 2015 Formoso et al., 2016	Mutations of glycine in TMD2 and TMD 4	Such mutations affect the formation of dimers and prevent them from inducing the filopodia formation [74, 75].
Alvarez Juliá et al., 2016	GPM6A inducing CORO1A to activate its downstream RAC1 and PAK1 signaling pathways	Promote filopodia formation [76].
Scorticati et al., 2011	Inhibition of Src kinase and MAPK	It prevents filopodia formation in M6a‐overexpressed neurons [33].

Abbreviations: CORO1A, coronin‐1a; M6a, N6‐methyladenosine; MAPK, mitogen‐activated protein kinase; PAK1, p21‐activated kinase 1; RAC1, Ras‐related C3 botulinum toxin substrate 1; TMDs, transmembrane domains.

**FIGURE 1:**
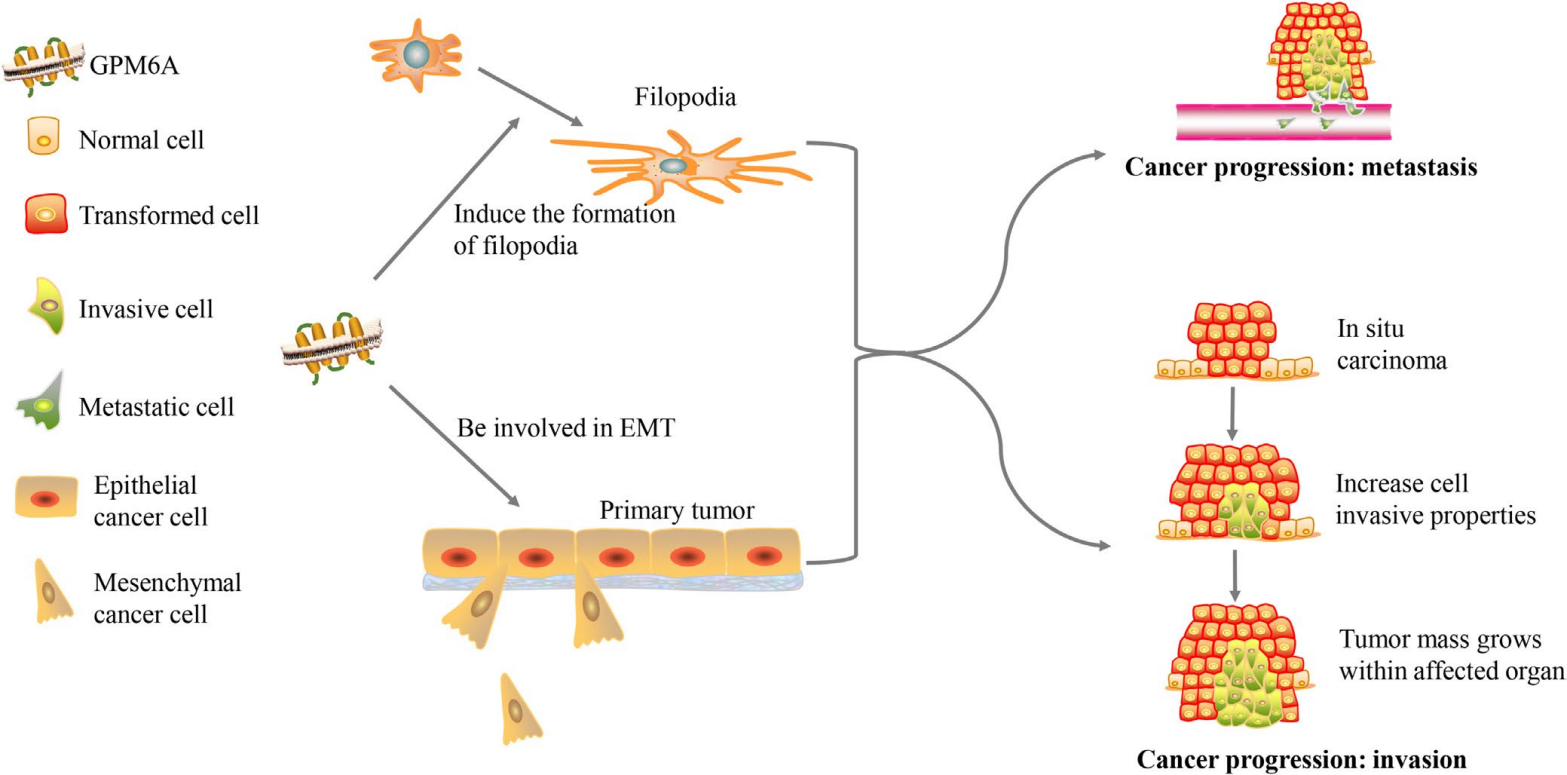
Possible mechanisms of GPM6A in malignant tumors.

GPM6A may make effect on the development of malignant tumors through influencing EMT and the formation of filopodia (Figure 1). In invasive GBSC, GPM6A is overexpressed and is localized in the lamellipodia/pseudopodia‐like structure, indicating that GPM6A plays a role in cell migration/invasion. Blocking the expression of GPM6A in GBSC spheres with specific siRNA can significantly reduce the invasiveness of the spheres. This proves that GPM6A is involved in the invasion of human tumor cells [15]. Decreased GPM6A expression may lead to the decrease of filopodia and the increase of lamellipodia, resulting in rapid metastasis of cancer cells, affecting the prognosis of lung cancer patients. Apart from that, GPM6A is negatively correlated with tumor volume doubling time (TVDT) [77]. The *GPM6A* gene may serve as a clinical prognostic indicator in malignant tumors.

## 4 Mechanisms of GPM6A in Malignant Tumors

### 4.1 Colorectal Cancer

GPM6A is closely related to the survival time of patients with rectal cancer and is a marker of poor prognosis of rectal cancer [78].

The most common type of CRC is colon adenocarcinoma (COAD) [79]. MiR‐133b is involved in human CRC progression by regulating the expression of CXCR4, and is a novel prognostic marker [80]. MiR‐133b and its seven target genes *GPM6A, GNB4, RUNX1T1, EPHA7, PTPRZ1, ADAMTS5*, and *BICC1* may be important molecular targets of COAD [81].

Missing oncogene *GPM6A* was found in over half of metastatic CRC (mCRC) patients who received chemotherapy [82]. The methylation of heart and neural crest derivatives‐expressed protein 2 (HAND2) is a common and critical molecular change in endometrial cancer, which may act as a biomarker for its early detection and serve as a predictor for therapeutic response [83]. In cervical cancer, abnormal HAND2 DNA methylation has been observed [84]. High‐frequency cancer‐specific methylation and silenced expression of HAND2, as well as low survival rate, were demonstrated in CRC [85]. Chromosome 2 open reading frame 40 (C2orf40), also known as esophageal cancer‐related gene 4 (ECRG4), a tumor suppressor, is hypermethylated in cancers such as colorectal carcinoma [86], glioma [86], breast cancer [87], gastric cancer [88], and bladder cancer [89]. Overexpression of *C2orf40* impairs colon cancer cell proliferation, while the re‐expression of the silenced *C2orf40* gene restores its inhibitory effect on colon cancer cell growth [86]. Abnormal gene methylation has been widely reported in malignant tumors, not only affecting carcinogenicity but also contributing to metastasis [86, 90, 91]. *HAND2*, *C2orf40*, and *GPM6A* are hypermethylated and downregulated in tumor tissues, but low expression of all the three genes is related to significantly prolonged survival, and their upregulation results in poor prognosis of CRC patients [16]. The function of these genes may differ during the development and progression of malignant tumors. Simultaneous decreased expression levels of HAND2, C2orf40, and GPM6A proteins were found in highly differentiated CRC tissues, while higher expression was observed in minimally differentiated or undifferentiated colon cancer tissues [16], suggesting that GPM6A is related to poor prognosis of malignant tumors. HAND2, C2orf40, and GPM6A have potential prognostic value, but their role in the development of CRC still needs further research.

### 4.2 Lung Cancer

The study of Chen et al. [92] pointed out that *GPM6A* may be a gene closely related to lung carcinoma, and its expression is downregulated in patients. The analysis of differences in m6A regulatory factors between the low‐risk and high‐risk groups showed that the low‐risk group had higher expression of GPM6A, FTO, YTHDC2, and METTL3 [93].

Small‐cell lung cancer (SCLC) is sensitive to radiotherapy, but it develops anti‐radioactivity in the later stage of radiotherapy, which leads to the recurrence of SCLC [18]. MicroRNAs (miRNAs) play a role in regulating gene expression during the occurrence and development of tumors [94, 95]. MiR‐22‐3p (abbreviated as miR‐22) has significant dose‐dependent effect on migration, proliferation, and apoptosis of SCLC cells [18]. MiR‐22 enhances SCLC radiosensitivity by targeting Werner Interacting Protein 1 (WRNIP1) [18]. Stanniocalcin 1 (STC1), a glycoprotein found in the endocrine glands of fish kidney [96], has enhanced expression in HCC [97], ovarian cancer [98], breast cancer [99], and GB [100]. STC1 promotes the viability and proliferation of tumor cells and enhances the invasion and metastasis of solid tumors [101]. Compared with the control group, the expressions of GPM6A and STC1 were significantly higher in miR‐22‐overexpressed cell lines, suggesting that GPM6A and STC1 may be apoptosis inducers in SCLC cells. MiR‐22 may contribute to the apoptosis of SCLC cells through increasing expression levels of the two apoptosis‐inducing factors in tumors [18], but the relationship between miR‐22 and GPM6A in SCLC requires further investigation.

Lung adenocarcinoma, the most common subtype of lung cancer, has high morbidity and mortality [17]. The expression of GPM6A in lung adenocarcinoma was lower than that in adjacent tissues or normal lung tissues, and similar result was observed in lung adenocarcinoma cells [17]. Functional studies have shown that upregulation of GPM6A inhibits the development of lung adenocarcinoma cells, whereas downregulation of GPM6A enhances their migration, proliferation, and EMT [17]. Xenograft results showed that GPM6A upregulation could delay tumor growth and reduce tumor weight [17]. In addition, Western blotting (WB) indicated that knocking down GPM6A activated PI3K/AKT pathway, while the upregulation GPM6A inhibited its activation [17]. PI3K/AKT pathway is aberrantly activated in cancers, contributing to the occurrence and progression of tumors [102]. In summary, GPM6A inhibits the progression of lung adenocarcinoma via inhibiting PI3K/AKT pathway. Apart from that, the MIR99AHG/miR‐218‐5p/GPM6A axis has a twofold lung cancer inhibitory effect in lung adenocarcinoma through the non‐coding tumor suppressor gene *MIR99AHG* [103, 104]. This axis has significant diagnostic and prognostic value for lung cancer patients. Thus, GPM6A may be a potential target for the treatment of human lung cancer [17].

### 4.3 Liver Cancer

Liu et al. [105] explored the mechanism of AKT/ERK signaling pathway and gene *DYNC1I1* regulating cell cycle through hsa_circ_0001495 (circCCNB1)/miR‐106b‐5p/GPM6A network. The expressions of GPM6A and circCCNB1 are downregulated significantly and miR‐106b‐5p expression is upregulated in carcinoma tissues and HCC cells. The silencing of circCCNB1 promotes the cloning ability, G1‐S cell cycle transition, and xenograft tumor growth of liver cancer cells by downregulating GPM6A. Low expression of GPM6A increases the expression of dynein cytoplasmic 1 intermediate chain 1 (DYNC1I1) and activates the phosphorylation of the threonine protein kinase (AKT)/MAPK 1 (ERK) pathway to regulate cell cycle in HCC [105].

GPM6A is a protective factor for patients with HCC [106]. Its expression is different in normal tissue and tumor tissue of patients, and is associated with prognosis [106]. GPM6A overexpression hinders cell proliferation, formation of colony, as well as migration and invasion of some types of liver cancer cells [19, 105]. The GPM6A levels found in HCC tissue are much lower than those in pericancerous liver tissue, and this is associated with poor prognosis [19]. In GPM6A‐overexpressed liver cancer cells, the function of oncogenic microRNA‐96 [107, 108, 109] was significantly inhibited [19]. Besides, GPM6A can induce apoptosis of HCC cells [106]. The interaction between GPM6A and microRNA‐96 in liver cancer requires additional validation.

GPM6A was found to activate the Smad pathway in HCC cells [106], which serves as an important negative regulatory signaling pathway for the proliferation of epithelial cells, regulates tumor cell apoptosis and proliferation [110]. In the early stage of tumor, the Smad signaling pathway inhibits cancer cell proliferation and induces apoptosis [111]. In the advanced stage of tumor, it promotes tumor progression via participating in immunosuppression, enhancing tumor cell invasion, and rebuilding the microenvironment [111]. For example, SMAD4 inhibits tumor progression initiated by KRAS (G12D) in pancreatic ductal adenocarcinomas (PDAC); in a subset of advanced tumor, intact SMAD4 promotes TGF‐β‐dependent growth and EMT [112]. The function of TGF‐β depends on the environment and cell type. The role of this signal transduction in tumor progression is twofold ambivalent [113, 114]: in healthy and early cancer cells, activation of the TGF‐β signaling leads to cell cycle arrest and apoptosis; in advanced malignancies, TGF‐β signaling pathway induces metastasis and drug resistance [115]. Therefore, the Smad signaling pathway such as TGF‐β‐SMAD signaling pathway plays a double‐edged role in the treatment of malignant tumors [112, 116], and it is context‐dependent. There are data suggesting that GPM6A inhibits liver cancer cells through the Smad signaling pathway, but the function of GPM6A still needs to be validated in vivo [106]. In summary, GPM6A may be a valuable biomarker for the progression and prognosis of HCC.

### 4.4 Glioblastoma

GB is an invasive brain tumor, whose invasiveness is fundamentally derived from tumor stem cells [117]. GBSC has the ability of self‐renewal, which helps the occurrence of tumors [15]. Despite surgery and chemoradiotherapy, the systemic recurrence of GB is due to GBSC, which is particularly aggressive [117] and radiation‐resistant [118]. Therefore, decoding the molecular mechanism of GBSC resistance and invasiveness is crucial for developing new effective treatments for GB [15].

**FIGURE 2:**
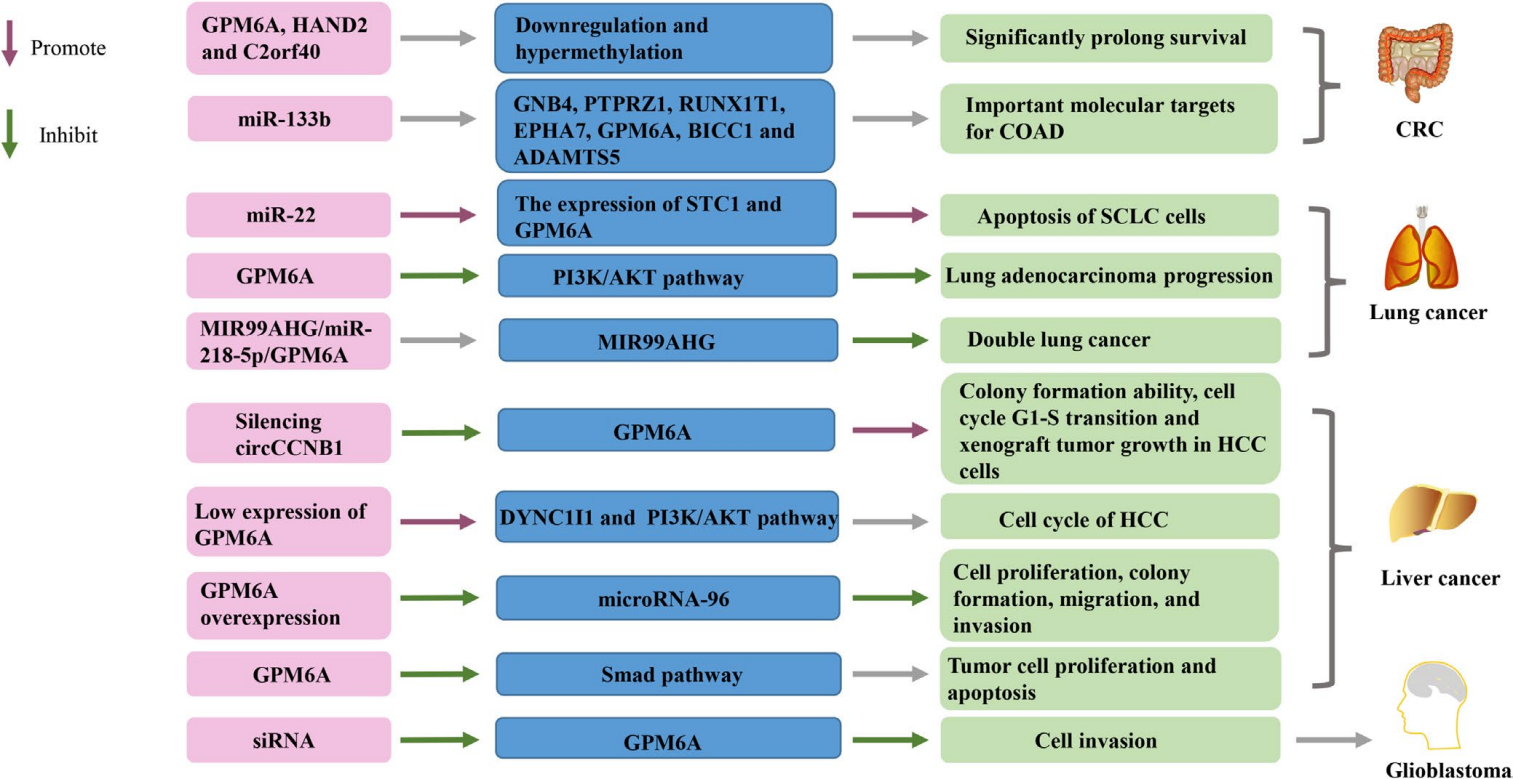
GPM6A in malignant tumors.

Compared to noninvasive cells, the expression of GPM6A was higher in invasive GBSC and it is localized in the pseudopodia/lamellipodia‐like structure [15]. Rich filopodia are related to enhanced metastasis and invasion of malignant tumor cells [55, 56, 57]. Blocking the expression of GPM6A with siRNA resulted in a significant reduction in cell invasion, indicating that GPM6A plays a role in the invasion of human tumor cells [15]. GPM6A expression is crucial for GBSC to form spheres as knocking down GPM6A significantly reduces neurospheres formed by GBSC derived from GB biopsy specimens [15]. Also, carcinogenic PTPRZ1 is highly expressed in GB and is related to tumorigenicity, participating in tumor cell invasion [119, 120]. Knocking down PTPRZ inhibits the migration and growth of GB in vitro and in vivo [121, 122]. The anticancer drug temozolomide (TMZ) used to treat GB interferes with DNA synthesis and subsequent cell cycle processes, thus inducing the death of cancer cells. However, cancer cells at quiescent stage do not require DNA synthesis, so they evade the anti‐cancer effects of TMZ. TMZ disrupts cell cycle‐induced cell death, becoming the culprit for GB recurrence. This process is achieved through the expression and activation of PTPRZ1 on the cancer stem cell (CSC) membrane [123]. It has been shown that targeting Wnt/β‐catenin signaling pathway can enhance the radiation response of glioma stem cells [124]. Downregulating PTPRZ1 and Wnt8a transcription makes pancreatic cancer cells radiation‐sensitive, thereby inhibiting the Wnt/β‐catenin signaling pathway [125]. GPM6A is highly correlated with PTPRZ1 expression in GB, and is positively correlated with ZEB1, an EMT‐transcription factor (EMT‐TF) that promotes cell invasion. GPM6A and PTPRZ1 are essential for GBSC sphere formation. When they are blocked by their respective specific siRNA, the number of spheres is significantly reduced, and blocking GPM6A or PTPRZ1 can increase the radiosensitivity of GBSC [15]. Blocking either GPM6A or PTPRZ1 may be an interesting method for treating GB because it could simultaneously target proliferation, invasion, and radiological resistance [15], but their potential as therapeutic targets should be validated through clinical studies. Further studies could explore how inhibiting or enhancing GPM6A activity might be leveraged in targeted therapies. For instance, a potential therapeutic strategy could be using RNA interference or small molecule inhibitors to modulate GPM6A expression (Figure 2).

### 4.5 Others

In addition to CRC, lung adenocarcinoma, liver cancer, and GB, there are other malignancies associated with *GPM6A* gene and its abnormal protein function. Exogenous expression of GPM6A induced unanchored cell growth and enhanced proliferation of NIH/3T3 cells [3]. In B‐type leukemia, GPM6A is dysregulated [126]. GPM6A isoform 3 is overexpressed in human B‐cell leukemia [3]. Human GPM6A is specifically overexpressed in chronic lymphocytic leukemia (CLL) and mantel cell lymphoma (MCL) [3]. *GPM6A* is specifically related to mouse and human B‐cell leukemia and may be a candidate biomarker for B‐lymphocyte malignancy, and this gene may represent a potential proto‐oncogene [3, 126]. Apart from that, GPM6A is a new associated protein in thyroid cancer, and its expression level in thyroid cancer is significantly lower than that in normal tissues [127]. GPM6A expression was higher in fast‐growing gonadotroph adenomas (GA) than in slow‐growing GA [77] (Table 3).

**TABLE 3:** Expressions and influences of GPM6A in various malignant tumors.

First author, year	Types of malignant tumors	Expression of GPM6A	Species	Mechanisms
Ye et al., 2021	CRC	Downregulated	Human	Low levels of *GPM6A* expression were associated with significantly longer survival [16].
Chen et al., 2015	Lung carcinoma	Downregulated	Human	GPM6A expression level in lung carcinoma patients is lower than that in normal samples, suggesting that it is a gene closely related to lung carcinoma [92].
Jiang et al., 2019	SCLC	Unknown	Human	MiR‐22 may promote apoptosis of SCLC cells by increasing the expression levels of STC1 and GPM6A in tumors [18].
Zhang et al., 2022	Lung adenocarcinoma	Downregulated	Human, mice	Upregulation of GPM6A can delay tumor growth and reduce tumor weight [17]. GPM6A inhibits lung adenocarcinoma progression by inhibiting the PI3K/AKT pathway [17].
Sweef et al., 2022			Human	MIR99AHG/miR‐218‐5p/GPM6A axis has twofold lung cancer inhibitory effect in lung adenocarcinoma [104].
Liu et al., 2022	HCC	Downregulated	Human	Low GPM6A expression increases the expression of DYNC1I1 and activates AKT/ERK pathway phosphorylation, thereby regulating HCC cell cycle [105].
Li et al., 2022	HCC	Downregulated	Human	In GPM6A‐overexpressed hepatoma cells, the function of microRNA‐96 was inhibited significantly [19].
Wen et al., 2023	HCC		Human	GPM6A activates Smad signaling pathway [106], and the role of this pathway in malignant tumors is ambiguous.
Lacore et al., 2022	GB	Upregulated	Human	GPM6A is expressed higher in invasive GBSC. PTPRZ1 regulates GPM6A expression and cell invasion, both of which are critical for GBSC to form spheres [15].
Charfi et al., 2014	Lymphoblastic leukemia	Upregulated	Human	Exogenous expression of GPM6A can promote the unanchored growth of NIH/3 T3 cells [3].
Charfi et al., 2014	MCL	Upregulated	Human	Human GPM6A is specifically overexpressed in MCL [3].
Khalid et al., 2012	Thyroid cancer	Downregulated	Human	Compared with normal tissue, GPM6A expression in thyroid cancer was significantly lower [127].
Falch et al., 2018	GA	Upregulated	Human	The expression of GPM6A was higher in rapidly growing GA [77].

Abbreviations: AKT, AKT serine; CRC, colorectal cancer; DYNC1I1, dynein cytoplasmic 1 intermediate chain 1; ERK, mitogen activated protein kinase 1; GA, gonadotroph adenomas; GB, glioblastoma; GBSC, GB stem cells; HCC, hepatocellular carcinoma; MCL, mantel cell lymphoma; PI3K, phosphoinositide 3‐kinase; PTPRZ1, protein tyrosine phosphatase receptor type Z1; SCLC, small‐cell lung cancer; STC1, Stanniocalcin 1.

## 5 Discussion

In some malignant tumors, GPM6A shows cancer‐promoting properties. GPM6A is specifically overexpressed in MCL and CLL [3]. *GPM6A* is specifically associated with B‐cell leukemia and may be its candidate biomarker. This gene may represent a potential proto‐oncogene [3, 126]. GPM6A is related to poor prognosis in patients with rectal cancer or CRC. Decreased expression of GPM6A protein was found in highly differentiated CRC tissues, while higher expression levels were observed in minimally differentiated or undifferentiated colon cancer tissues [16], but its relationship with CRC is still unclear. The expression of GPM6A is crucial for GBSC to form spheres, and blocking its expression significantly reduces cell invasion [15]. The expression of GPM6A is higher in fast‐growing GA [77]. These suggest that GPM6A may be a procancerous factor in malignant tumors.

However, GPM6A exhibits the characteristics of tumor suppressor genes in other malignant tumors. GPM6A may be a cell apoptosis inducing factor. MiR‐22 may enhance SCLC cell apoptosis via increasing the expression of GPM6A and STC1 in tumors [18]. GPM6A upregulation inhibits the development of lung adenocarcinoma cells, delays tumor growth, and reduces tumor weight. It inhibits lung adenocarcinoma progression through inhibiting the PI3K/AKT signaling pathway [17]. MIR99AHG/miR‐218‐5p/GPM6A axis has twofold lung cancer inhibitory effect in lung adenocarcinoma [104]. GPM6A expression in thyroid cancer is significantly lower than that in normal tissues [127]. These suggest that GPM6A may be an inhibitory factor for malignant tumors.

In addition, the role of GPM6A in certain malignant tumors needs further clarification. Overexpression of GPM6A decreases the proliferation of liver cancer cells, colony formation, invasion, and migration [19, 105], inhibits the carcinogenic function of microRNA‐96 [19], and can also induce apoptosis of HCC cells [106]. Nevertheless, GPM6A was found to activate the Smad pathway in HCC cells [106], while the Smad signaling pathway is a double‐edged sword for promoting and suppressing cancer in the treatment of malignant tumors [112, 116]. Although there is data indicating that GPM6A inhibits liver cancer cells through the Smad pathway, its function needs to be validated in vivo [106].

In summary, the role of GPM6A is controversial not only in different types of malignant tumors, but also in the same type of malignant tumors. Further clarification is needed to determine whether its controversial role in malignant tumors is directly related to its impact on EMT and induction of filopodia.

## 6 Conclusions and Prospects

Current studies have shown that GPM6A does participate in tumor occurrence and development, but what role it plays and how it regulates are still not clear. Downregulation of GPM6A enhanced the EMT of lung adenocarcinoma cells and EMT is necessary for most malignant tumor metastasis. GPM6A induces the formation of filopodia, which is involved in the adhesion and migration of cancer cells. *GPM6A* is a highly expressed potential oncogene in lymphoid leukemia. It has shown carcinogenic properties in CRC, GB, GA, and so on. However, the expression of *GPM6A* decreases in lung adenocarcinoma, liver cancer, thyroid cancer, and so forth as the tumor progresses, indicating that it may be a tumor suppressor gene. Although GPM6A is downregulated in tumors, its precise role in tumorigenesis needs further research. While GPM6A may have potential carcinogenic properties, additional evidence is needed to confirm this. An oncogene or a tumor suppressor? It depends on the malignancy and context. Potential roles of GPM6A in physiological functions are areas needing further investigation. More extensive studies are necessary to validate conclusions and to better understand the role of GPM6A in various cancers. Uncovering the underlying molecular mechanism of how GPM6A further affects the development and progression of malignant tumors by affecting EMT and filopodia will help elucidate the controversial role of GPM6A in malignant tumors. Further understanding of the structure and function of its SNPs will help to better understand the relationship between GPM6A and various malignant tumors. Although the biological function of GPM6A in the development of malignant tumors is still unclear, according to the current research progress, it is still expected to become an effective molecular marker for predicting tumor occurrence, metastasis, and prognosis, as well as a new target for diagnosis and treatment. Further researches are needed to confirm and elaborate on the observed roles of GPM6A in various cancer processes and its potential as a prognostic indicator.

## Author Contributions

Bei Huang drafted manuscript and prepared tables and figures. All figures were drawn by ourselves. Xihong Li revised the manuscript. Bei Huang and Xihong Li approved final version of manuscript. All authors critically reviewed and approved the final manuscript.

## Ethics Statement

The authors have nothing to report.

## Consent

The authors have nothing to report.

## Conflicts of Interest

The authors declare no conflicts of interest.

## Data Availability

The authors have nothing to report.
